# Prognostic Significance of SGK1 Expression in Multiple Myeloma Patients Undergoing Autologous Hematopoietic Stem Cell Transplantation: A Single‐Center Retrospective Study

**DOI:** 10.1155/sci/6738118

**Published:** 2026-01-30

**Authors:** Xiangdong Shen, Haiyan Liu, Xiaoyu Huang, Qiaocheng Qiu, Yuhua Ru, Hui Hui, Juncheng Chen

**Affiliations:** ^1^ Department of Hematology, Affiliated Hospital of Nantong University, No. 20 Xisi Road, Nantong, 226001, Jiangsu, China, ahnmc.com; ^2^ National Clinical Research Center for Hematologic Diseases, Jiangsu Institute of Hematology, The First Affiliated Hospital of Soochow University, No. 188 Shizi Street, Suzhou, 215006, Jiangsu, China, sdfyy.cn; ^3^ Institute of Blood and Marrow Transplantation, Collaborative Innovation Center of Hematology, Soochow University, Suzhou, China, suda.edu.cn; ^4^ Department of Hematology, Naval Medical Center of the People’s Liberation Army of China, No. 338 Huaihai Road, Changning District, Shanghai, 200052, China; ^5^ Department of Hematology, Jiangyin People’s Hospital Affiliated to Nantong University, No. 163 Shoushan Road, Jiangyin, 214400, Jiangsu, China

**Keywords:** autologous hematopoietic stem cell transplantation, biomarker, minimal residual disease, multiple myeloma, relapse prediction, ROC analysis, serum free light chain, SGK1

## Abstract

**Purpose:**

This research attempts to assess the prognostic significance of serum/glucocorticoid‐regulated kinase 1 (SGK1) expression in peripheral blood mononuclear cells (PBMCs) of multiple myeloma (MM) individuals undergoing autologous hematopoietic stem cell transplantation (AHSCT) compared to traditional minimal residual disease (MRD) and serum free light chain (sFLC) assessments.

**Methods:**

A single‐center, retrospective study was carried out involving 85 MM individuals who underwent AHSCT. SGK1 gene expression was measured in PBMCs using quantitative real‐time PCR (qRT‐PCR) at baseline and at defined post‐transplant intervals. Concurrently, MRD status was assessed using multiparameter flow cytometry (MFC) and sFLC levels were measured. Individuals were seen for a median of 36 months post‐transplant. ROC curve analysis was employed to assess the predictive power of SGK1 expression, MRD, and sFLC for relapse.

**Results:**

SGK1 gene expression demonstrated dynamic changes in AHSCT, with levels decreasing in all risk groups, reflecting reductions in disease burden. Quantitative analysis showed that the predictive efficacy of SGK1, utilizing the area under the receiver operating characteristic (ROC) curve (area under the curve [AUC]), was highly comparable to that of MRD assessments, with SGK1 achieving an AUC of 0.86, closely approximating the MRD AUC of 0.88. Persistent high SGK1 expression, particularly discernible in individuals harboring high‐risk (HR) cytogenetic profiles, was considerably associated with an elevated risk of relapse (hazard ratio for high vs. low SGK1 expression: 2.7; 95% CI: 1.4–5.3; *p*  < 0.01).

**Conclusion:**

SGK1 gene expression in PBMCs serves as a promising, minimally invasive biomarker for relapse prediction in MM individuals undergoing AHSCT.

## 1. Introduction

Multiple myeloma (MM) is a type of cancer originating from plasma cells that multiply uncontrollably within the bone marrow. This hyperproliferation results in significant organ damage, characterized by the CRAB criteria: bone lesions, anemia, renal failure, and hypercalcemia [1, 2]. Despite advances in the understanding and treatment of MM over recent decades, the disease remains incurable for most patients, with a pattern of repeated relapses and progression [3]. Autologous hematopoietic stem cell transplantation (AHSCT), along with the use of monoclonal antibodies, immunomodulatory agents, and proteasome inhibitors, forms the foundation of therapy for suitable patients, enhancing both progression‐free survival (PFS) and overall survival (OS) [4, 5]. However, disease relapse remains a major clinical challenge even after AHSCT, necessitating more precise tools for early prediction of relapse and assessment of residual disease burden.

Minimal residual disease (MRD) detection has emerged as a powerful prognostic marker in MM [6]. The use of multiparameter flow cytometry (MFC), next‐generation sequencing (NGS), and other sensitive techniques allows for the identification of residual malignant cells below the detection limit of conventional morphology [7, 8]. Achieving MRD negativity has been consistently associated with prolonged PFS and OS across multiple clinical trials and real‐world studies. However, limitations remain: MRD detection is dependent on bone marrow sampling, which is invasive and may not reflect systemic disease, particularly in cases with extramedullary involvement or patchy marrow infiltration [9]. Moreover, MRD negativity does not guarantee long‐term remission, highlighting the need for complementary biomarkers that can enhance risk stratification and guide post‐transplant surveillance.

Serum free light chain (sFLC) assay, another commonly used marker in MM, measures unbound *κ* and *λ* light chains in serum, offering a surrogate indicator of disease activity [10]. Although sFLC is more accessible than bone marrow‐based MRD assessment, it lacks sufficient specificity and sensitivity, particularly in patients with nonsecretory or oligosecretory disease. Consequently, while sFLC contributes valuable information, it is often used in conjunction with other parameters for relapse monitoring [11].

In recent years, increasing attention has turned toward the molecular profiling of MM to uncover novel prognostic indicators and therapeutic targets. Among these, serum/glucocorticoid‐regulated kinase 1 (SGK1) has emerged as a candidate biomarker with potential prognostic relevance in hematological malignancies [12]. SGK1 is a serine/threonine protein kinase regulated by glucocorticoids, serum, and cellular stress signals. Functionally, SGK1 plays roles in cell survival, ion transport, transcriptional regulation, and tumor cell proliferation [13–15]. In several cancer types, aberrant SGK1 expression has been linked to chemoresistance, enhanced survival signaling, and poor clinical outcomes. In hematologic malignancies, such as chronic lymphocytic leukemia (CLL) and acute myeloid leukemia (AML), elevated levels of SGK1 have been linked to both advanced disease stages and decreased responsiveness to treatment. However, its role in MM remains incompletely elucidated.

Preliminary evidence implies that SGK1 might lead to the survival of malignant plasma cells and the development of drug resistance, potentially influencing disease progression and relapse. Given its biological function and regulatory pathways—many of which intersect with known MM pathophysiology—it is plausible that SGK1 expression may serve as a prognostic marker in MM, particularly in the post‐transplant setting where relapse surveillance is crucial [16]. However, few clinical studies to date have systematically evaluated SGK1 expression in MM patients undergoing AHSCT or assessed its predictive value for relapse in comparison with established MRD measures.

To address this knowledge gap, we carried out a single‐center, retrospective study involving 85 MM individuals who underwent AHSCT and received standardized induction and maintenance regimens. Our research sought to ascertain the prognostic significance of SGK1 gene expression in peripheral blood mononuclear cells (PBMCs) at baseline and at defined post‐transplant intervals, using quantitative real‐time PCR (qRT‐PCR) as the detection method. SGK1 expression levels were normalized against GAPDH as an internal control, and dynamic changes were analyzed longitudinally. Concurrently, we monitored MRD status via MFC with a sensitivity threshold of 10^−6^, and assessed sFLC levels using established immunoassays. Patients were followed for a median duration of 36 months post‐transplant, during which clinical outcomes, including relapse, disease progression, PFS, and OS, were recorded.

The study cohort was divided into three cytogenetic risk groups—high, intermediate, and low—based on fluorescence in situ hybridization (FISH) analysis of chromosomal abnormalities. High‐risk (HR) features included del(17p), t(4;14), t(14;16), and 1q21 amplification. Treatment regimens were standardized across groups, consisting of induction with VRD (bortezomib, lenalidomide, and dexamethasone), PAD (bortezomib, doxorubicin, and dexamethasone), or VTD (bortezomib, thalidomide, and dexamethasone), then by high‐dose melphalan (200 mg/m^2^) conditioning and post‐transplant maintenance with lenalidomide or lenalidomide plus bortezomib. This homogeneity in treatment approach provided a consistent clinical background for evaluating the prognostic value of SGK1 expression.

We hypothesized that SGK1 expression would exhibit dynamic alterations post‐AHSCT and that higher expression levels would correlate with increased risk of relapse and shorter PFS. Furthermore, we postulated that SGK1 expression might offer predictive power comparable to or exceeding that of traditional MRD assessment and sFLC monitoring, particularly in cases where MRD remains undetectable. We evaluated the sensitivity, specificity, and area under the curve (AUC) for each parameter in predicting relapse using receiver operating characteristic (ROC) curve analysis to test these assumptions.

The overarching objective of this study was to validate SGK1 expression as a novel, minimally invasive biomarker for relapse surveillance in MM patients post‐AHSCT. By integrating SGK1 expression data with established MRD and sFLC monitoring protocols, we aimed to develop a more robust risk assessment framework to guide clinical decision‐making, such as the intensification of maintenance therapy or early intervention in HR individuals. Given the persistent challenge of relapse in MM, particularly in HR cytogenetic subgroups, our outcomes might offer new insights into the molecular determinants of disease recurrence and pave the way for personalized, biomarker‐driven management strategies in the post‐transplant setting.

## 2. Patients and Methods

### 2.1. Study Design and Patient Cohort

This retrospective, single‐center study was carried out at the Department of Hematology, Affiliated Hospital of Nantong University, and included a total of 85 individuals diagnosed with MM who underwent AHSCT between January 2018 and December 2021. All individuals were diagnosed based on the International Myeloma Working Group (IMWG) criteria and underwent standardized therapy for induction, conditioning, and maintenance depending upon the individual institution’s protocols. The Institutional Review Board of the Affiliated Hospital of Nantong University approved this retrospective study. Since this is a retrospective study, the institution does not have a specific code of approval number. Because of the retrospective nature of the study, informed consent was not required. All actions taken were in accordance with the basic ethical principles of the Declaration of Helsinki.

### 2.2. Inclusion and Exclusion Criteria

Inclusion criteria: (1) confirmed diagnosis of MM based on IMWG criteria; (2) age 18–70 years at the time of transplantation; (3) completion of standard induction therapy followed by AHSCT; (4) availability of serial peripheral blood and bone marrow samples for biomarker analysis; and (5) minimum follow‐up duration of 24 months. Exclusion criteria: (1) primary refractory disease; (2) previous allogeneic stem cell transplantation; (3) incomplete clinical data; and (4) presence of concurrent malignancies or significant comorbidities precluding regular follow‐up.

### 2.3. Treatment Protocol

All subjects underwent standard initial treatments of either bortezomib–thalidomide–dexamethasone, bortezomib–lenalidomide–dexamethasone, or bortezomib–doxorubicin–dexamethasone across 4–6 cycles. This was followed by a high‐dose melphalan conditioning regimen (200 mg/m^2^) in preparation for AHSCT. Mobilization of peripheral blood stem cells was achieved using granulocyte colony‐stimulating factor (G‐CSF), with cyclophosphamide used optionally.

### 2.4. Risk Stratification

Patients were divided into three cytogenetic risk categories (HR, moderate‐risk [MR], and low‐risk [LR]) based on FISH analysis of bone marrow aspirates. Among the HR cytogenetic abnormalities were 1q21 gain, t(4;14), t(14;16), and del(17p). Patients lacking these abnormalities were considered to have standard‐risk cytogenetics.

### 2.5. Sample Collection and Biomarker Assessment

PBMCs were gathered at pre‐established intervals: before transplantation (baseline), and 3, 6, 12, and 24 months afterwards. Ficoll‐Paque density gradient centrifugation was used to separate the cells, which were subsequently kept at −80°C for further examination.

### 2.6. SGK1 Expression Analysis

Total RNA was extracted from PBMCs using the TRIzol reagent (Invitrogen, USA), and the RNA was then converted to cDNA using a High‐Capacity cDNA Reverse Transcription Kit (Applied Biosystems, USA). The SYBR Green Master Mix (Takara Bio, Japan) was used in qRT‐PCR to assess the amounts of SGK1 mRNA using the StepOnePlus real‐time PCR system (Applied Biosystems). GAPDH was utilized as the internal reference gene for normalization. GAPDH was selected because its expression remained stable across PBMC samples in our cohort and has been validated in previous MM studies as a reliable reference gene.

### 2.7. MRD

MRD evaluation utilized 10‐color MFC on bone marrow samples, achieving detection sensitivity down to 10^−6^. The criteria for MRD negativity were the nondetection of clonotypic plasma cells in a minimum of 1,000,000 evaluated events. The testing was conducted in the hematology laboratory of the institution by skilled cytometrists who were not informed of the patients’ clinical results.

### 2.8. sFLC Assay

Using a nephelometric platform, the Freelite assay (Binding Site Group, UK) was used to quantify serum κ and *λ* free light chains. The distinction between involved and uninvolved light chains (dFLC) was used as a measure of disease activity. A dFLC reduction ≥90% from baseline was considered indicative of response.

### 2.9. Clinical Data Collection and Outcomes

Demographic characteristics, laboratory values, cytogenetic data, treatment details, and response parameters were collected from electronic medical records. Disease response and progression were described in accordance with the IMWG criteria. Follow‐up evaluations were conducted at regular intervals with clinical examination, laboratory testing, imaging, and bone marrow assessments as clinically indicated.

The primary clinical outcomes measured were PFS and OS. PFS was measured from the time of transplantation until disease progression or death due to any cause, whereas OS was calculated from the time of transplantation until death from any cause. Additionally, instances of relapse were documented and analyzed in relation to SGK1 expression levels, MRD status, and sFLC levels.

### 2.10. Statistical Analysis

For statistical analyses, SPSS version 26.0 (IBM Corp., Armonk, NY, USA) and GraphPad Prism version 9.0 (GraphPad Software, USA) were utilized. Categorical data were compared using chi‐squared tests or Fisher’s exact tests. When appropriate, one‐way ANOVA or independent *t*‐tests were used to analyze continuous data. Changes in biomarker levels over time were analyzed using repeated measures ANOVA. The Kaplan–Meier method was used to determine survival rates, and the log‐rank test was used to examine group differences. The ROC curve analyses assessed the prognostic validity of SGK1 expression, MRD status, and sFLC levels to predict recurrence events. We calculated the AUC, sensitivity, and specificity for each indicator. A priori power analysis was not conducted; post hoc calculations confirmed the study had >80% power to accurately identify a hazard ratio ≥2.5 for relapse with SGK1 stratification at *α* = 0.05. A *p*‐value of <0.05 was considered statistically significant for all tests.

## 3. Results

### 3.1. Baseline Characteristics of Patients by Risk Group

Eighty‐five patients were analyzed as described above to identify 20 HR, 60 MR, and five LR patients. Demographic distribution and baseline characteristics are detailed in Table 1. There were no differences among groups for sex (*p* = 0.85), age (median 61, 59, and 56 for HR, MR, and LR, respectively; *p* = 0.74), or weight (71, 73, and 70 kg, *p* = 0.87). Similarly, we did not see significant differences for smoking history, hypertension, diabetes, and coronary artery disease (all *p*  > 0.40).

**Table 1:** Baseline characteristics of patients by risk groups.

Baseline characteristics	High‐risk group (*n* = 20)	Moderate‐risk group (*n* = 60)	Low‐risk group (*n* = 5)	*p*‐Value
Sex				
Male (%)	52% (10/20)	58% (35/60)	60% (3/5)	0.85
Female (%)	48% (10/20)	42% (25/60)	40% (2/5)	
Age (years)	61 (56–66)	59 (53–65)	56 (51–61)	0.74
Weight (kg)	71 (66–76)	73 (68–78)	70 (64–75)	0.87
Smoking history (%)	26% (5/20)	22% (13/60)	20% (1/5)	0.82
History of hypertension (%)	34% (7/20)	23% (14/60)	20% (1/5)	0.56
Diabetes history (%)	21% (4/20)	17% (10/60)	20% (1/5)	0.78
Coronary artery disease (%)	12% (2/20)	7% (4/60)	0% (0/5)	0.43
Liver dysfunction (ALT/AST >2 × ULN, %)	12% (2/20)	8% (5/60)	0% (0/5)	0.33
Renal impairment (Cr ≥2 mg/dL, %)	11% (2/20)	8% (5/60)	0% (0/5)	0.29
Peripheral blood WBC (× 10^9^/L)	5.4 (4.7–5.9)	5.6 (4.8–6.2)	5.9 (5.3–6.1)	0.65
Peripheral blood hemoglobin (g/dL)	11.3 (10.9–11.6)	11.7 (11.1–12.2)	12.1 (11.6–12.6)	0.72
Peripheral blood platelet count (× 10^9^/L)	152 (123–179)	162 (134–192)	172 (151–201)	0.68
Peripheral blood BUN (mg/dL)	19 (17–22)	18 (16–21)	17 (15–19)	0.75
Peripheral blood creatinine (Cr ≥2 mg/dL, %)	1.3 (1.1–1.6)	1.2 (1.0–1.4)	1.1 (0.9–1.2)	0.81
Comorbidities	1.6 (0–3)	1.2 (0–3)	1 (0–2)	0.90
Bone marrow plasma cell percentage (%)	41 (31–51)	36 (31–46)	31 (21–41)	0.55
Pre‐op liver function (ALT, IU/L)	36 (31–51)	31 (26–41)	26 (21–31)	0.62
Pre‐op liver function (AST, IU/L)	39 (36–56)	33 (29–46)	28 (23–34)	0.60
Pre‐op total bilirubin (mg/dL)	0.9 (0.8–1.2)	0.8 (0.7–1.0)	0.7 (0.6–0.9)	0.70
Pre‐op albumin (g/dL)	3.9 (3.6–4.1)	4.1 (3.9–4.3)	4.3 (4.1–4.5)	0.73

Liver and renal function (i.e., ALT/AST >2 × ULN, *p* = 0.33; creatinine ≥2 mg/dL, *p* = 0.29) were not different among groups, indicating similar liver and renal function at baseline. Indicators of peripheral blood (WBC, hemoglobin, platelet count, BUN, and creatinine) were also not statistically different among groups (all *p*  > 0.60). Baseline liver function markers are now reported as separate values (ALT, AST, total bilirubin, and albumin) to avoid redundancy in the presentation.

The mean number of comorbidities for HR, MR, and LR was 1.6, 1.2, and 1.0, respectively (*p* = 0.90). The percentage of bone marrow plasma cells and liver function measures were not different among groups (*p* = 0.55–0.73). These results verify balanced baseline clinical and laboratory characteristics across patients in the three groups.

### 3.2. Clinical Indicators and Treatments by Risk Groups

Clinical indicators and treatments are summarized in Table 2. Compared to the MR and LR groups, the HR group had a significantly higher percentage of IgG subtype (*p* = 0.045). The distributions of ISS staging were differentially significant, with a higher distribution of Stage III patients in the HR group (*p* = 0.027), while a higher distribution of Stage I patients was hospitalized in the LR group (*p* = 0.009). The HR group also had a higher percentage of adverse cytogenetic abnormalities, in addition to the del(17p) finding (*p*  < 0.001). The preoperative M protein and sFLC differences were significantly higher in the HR group compared to the MR and LR groups (both *p*  < 0.001).

**Table 2:** Clinical indicators and treatment approaches.

Clinical indicator/treatment	High‐risk group (*n* = 20)	Intermediate‐risk group (*n* = 60)	Low‐risk group (*n* = 5)	*p*‐Value
Immunoglobulin subtype				
IgG type	68% (14/20)	52% (31/60)	58% (3/5)	0.045
IgA type	16% (3/20)	22% (13/60)	18% (1/5)	0.738
Light chain type	16% (3/20)	26% (16/60)	24% (1/5)	0.623
ISS stage				
Stage III	62% (12/20)	28% (17/60)	22% (1/5)	0.027
Stage I	12% (2/20)	32% (19/60)	58% (3/5)	0.009
Stage II	26% (6/20)	40% (24/60)	20% (1/5)	0.112
High‐risk chromosomal abnormalities				
17p Deletion	26% (5/20)	7% (4/60)	0% (0/5)	<0.001
t(4;14)	13% (3/20)	9% (6/60)	0% (0/5)	0.342
Preoperative M protein level (g/dL)	2.1 (1.9–2.4)	1.4 (1.2–1.9)	1.3 (1.1–1.5)	<0.001
Preoperative sFLC difference (mg/L)	255 (210–290)	210 (160–240)	155 (110–175)	<0.001
Treatment regimen				
VTD regimen	48% (10/20)	44% (26/60)	42% (2/5)	0.860
VRD regimen	32% (6/20)	37% (22/60)	60% (3/5)	0.184
PAD regimen	20% (4/20)	19% (11/60)	0% (0/5)	0.157
High‐dose melphalan (200 mg/m^2^)	100% (20/20)	100% (60/60)	100% (5/5)	1.000
Post‐transplant maintenance treatment				
Lenalidomide maintenance	58% (12/20)	51% (31/60)	42% (2/5)	0.712
Bortezomib + lenalidomide maintenance	42% (8/20)	49% (30/60)	60% (3/5)	0.321

### 3.3. Treatment and Outcomes of Transplant and Follow‐Up Periods

Descriptive treatment outcomes are shown in Table 3 and Figure 1.

Figure 1Outcomes of transplant treatment and follow‐up periods. (A) Treatment responses (CR, PR, ORR, SD, and PD) across risk groups. (B) Cumulative relapse/progression at 3‐, 6‐, 12‐, and 24‐month post‐transplant.

**Figure:**
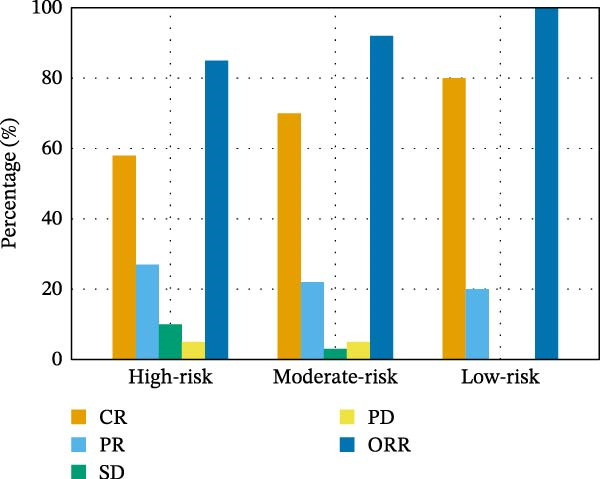
(A)

**Figure:**
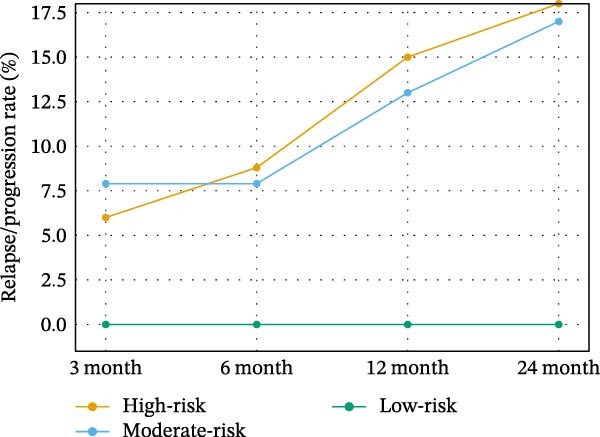
(B)

**Table 3:** Outcomes of transplant treatment and follow‐up periods.

Treatment response/follow‐up time	High‐risk group (*n* = 20)	Moderate‐risk group (*n* = 60)	Low‐risk group (*n* = 5)
Complete remission (CR)	58% (11/20)	70% (42/60)	80% (4/5)
Partial remission (PR)	27% (5/20)	22% (13/60)	20% (1/5)
Overall response rate (ORR) (CR + PR)	85% (17/20)	92% (55/60)	100% (5/5)
Stable disease (SD)	10% (2/20)	3% (2/60)	0% (0/5)
Disease progression (PD)	5% (1/20)	5% (3/60)	0% (0/5)
Relapse/progression after 3 months	6% (1/20)	8% (5/60)	0% (0/5)
Relapse/progression after 6 months	9% (2/20)	8% (5/60)	0% (0/5)
Relapse/progression after 12 months	15% (3/20)	13% (8/60)	0% (0/5)
Relapse/progression after 24 months	18% (4/20)	17% (10/60)	0% (0/5)

Outcomes in the HR (*n* = 20) group demonstrated a CR of 58% (11/20) and PR of 27% (5/20), for an ORR of 85%. SD and PD were noted in 10% and 5%, respectively, while 6%, 9%, 15%, and 18% of patients relapsed/progressed at 3‐, 6‐, 12‐, and 24‐month post‐transplant, respectively.

In the MR (*n* = 60) group, the ORR was 92% with 73% (44/60) recording CR and 22% (13/60) recording PR. SD and PD were recorded in 3% and 5%, respectively. The rate of relapse/progression at 3 months was 8%, at 6 months it was 8%, at 12 months it was 13%, and at 24 months it was 17%. In the LR group (*n* = 5), the ORR of 100% (CR: 80%; PR: 20%) was remarkable, and there were no cases of SD, PD, or relapse/progression at the 24‐month follow‐up.

Figure 1A shows treatment responses (CR/PR/SD/PD) on the *y*‐axis from baseline to 24 months by risk group, and Figure 1B shows cumulative relapse/progression rates at 3, 6, 12, and 24 months.

### 3.4. Dynamic Changes in MRD and SGK1 Expression Over Time Points

Some dynamic changes in MRD, sFLC, and SGK1 expression are reported in Table 4 and Figure 2. MRD positivity decreased from a baseline 30%–11% at the 24‐month follow‐up. sFLC levels were parallel to this decline (362 mg/L at baseline and 85 mg/L at 24 months), as was SGK1 expression (ΔCt 8.4–4.7).

Figure 2Dynamic changes in (A) MRD positivity proportion, (B) sFLC levels, and (C) SGK1 expression (ΔCt values) over time from baseline to 24 months. Statistical significance vs. baseline is shown as  ^∗^ *p*  < 0.05,  ^∗∗^ *p*  < 0.01,  ^∗∗∗^ *p*  < 0.001.

**Figure:**
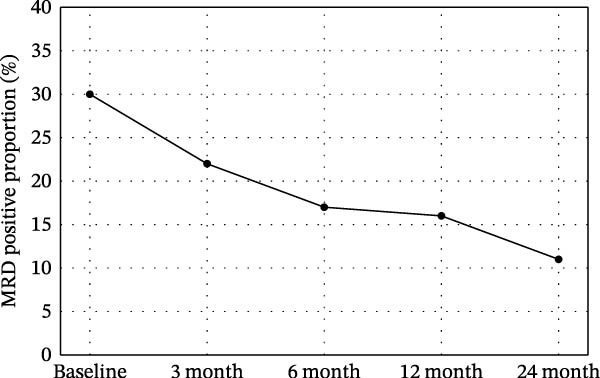
(A)

**Figure:**
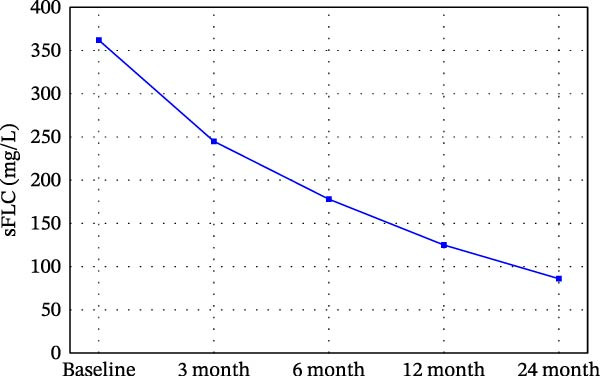
(B)

**Figure:**
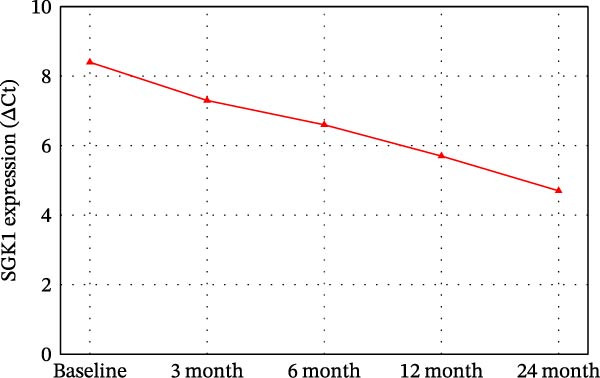
(C)

**Table 4:** Dynamic changes in MRD and SGK1 expression over follow‐up time points.

Time point	MRD positive proportion (flow cytometry)	sFLC (mg/L)	SGK1 gene expression (ΔCt value)
Baseline (pre‐transplant)	30% (25/85)	362 (305–413)	8.4 (7.9–8.8)
3 months post‐transplant	22% (19/85)	243 (215–276)	7.3 (6.8–7.9)
6 months post‐transplant	17% (14/85)	178 (156–215)	6.6 (6.2–7.1)
12 months post‐transplant	16% (14/85)	123 (105–142)	5.7 (5.2–6.3)
24 months post‐transplant	11% (9/85)	85 (67–98)	4.7 (4.3–5.2)

^∗∗^SGK1 expression, which had a consistent downward trajectory, paralleled both MRD and sFLC improvements in the present study and likely serves as a biomarker of disease burden. Figure 2B,C now denotes statistical significance versus baseline using the asterisk notation ( ^∗^ *p*  < 0.05,  ^∗∗^ *p*  < 0.01,  ^∗∗∗^ *p*  < 0.001).

Exploratory profiling of cytokines from PBMCs was not performed. However, issues regarding SGK1’s likelihood of a cytokine response are discussed in the discussion section (limitations/future work).

### 3.5. Comparing Predictive Efficacy of MRD, sFLC, and SGK1 for Relapse Prediction

Table 5 and Figure 3 describe predictive efficacy as a comparison. For MRD using flow cytometry, there was the best AUC (0.88, 95% CI: 0.84–0.92; sensitivity 85%, specificity 80%). SGK1 expression has similar predictive efficacy (AUC 0.86, 95% CI: 0.81–0.91; sensitivity 80%, specificity 82%) and is better than sFLC (AUC 0.80, 95% CI: 0.75–0.85; sensitivity 75%, specificity 78%).

Figure 3Prognostic and predictive analyses of SGK1 expression. (A) Kaplan–Meier progression‐free survival (PFS) curves stratified by SGK1 high vs. low groups. (B) Kaplan–Meier overall survival (OS) curves stratified by SGK1 high vs. low groups. (C) ROC curve comparing predictive efficacy of MRD, sFLC, and SGK1 expression for relapse.

**Figure:**
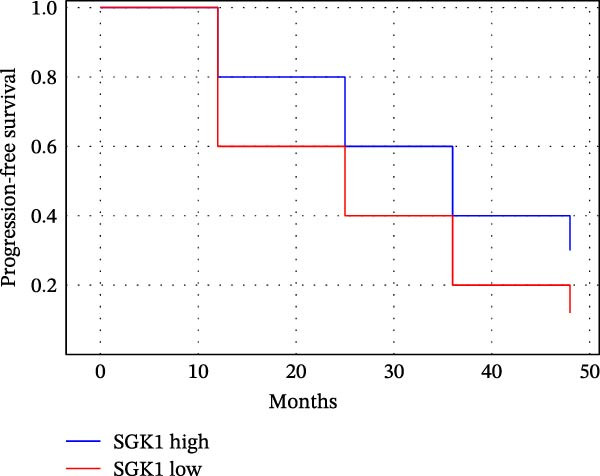
(A)

**Figure:**
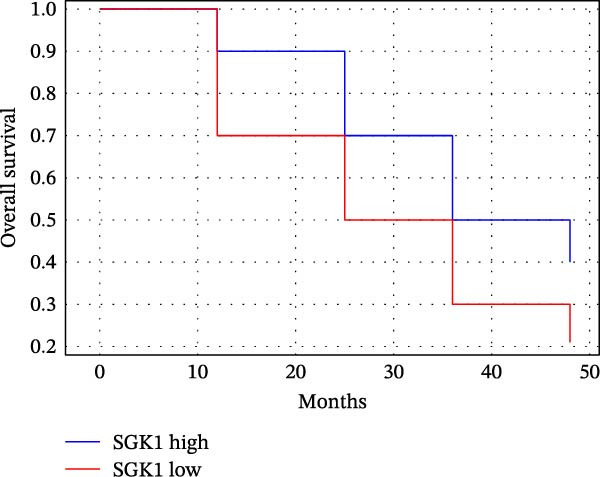
(B)

**Figure:**
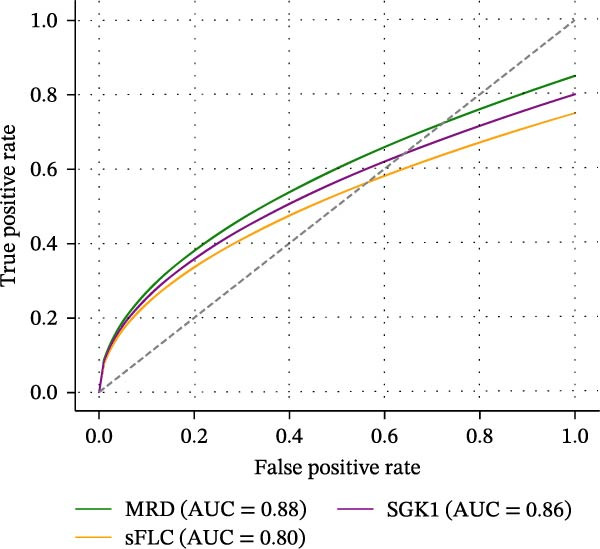
(C)

**Table 5:** Comparative predictive efficacy of existing MRD markers vs. SGK1 gene markers for relapse prediction.

Indicator	AUC	95% CI for AUC	Sensitivity (%)	Specificity(%)
MRD (flow cytometry)	0.88	0.84–0.92	85	80
sFLC (serum free light chains)	0.80	0.75–0.85	75	78
SGK1 gene expression	0.86	0.81–0.91	80	82

The variability predictive efficacy was compared at a threshold for the ΔCt of SGK1, using Youden’s index (ΔCt = 6.1), with the same results at a threshold of the sensitivity/specificity of 80%–82%.

The Kaplan–Meier analyses (Figure 3A,B) confirmed the prediction with significance in patients with higher expression of SGK1 with PFS, with a median 18.5 months vs. not reached; log‐rank *p* = 0.004, and a trend for OS, median 32.1 months vs. not reached; log‐rank *p* = 0.081. Hazard ratios for relapse/progression for the high‐SGK1 group were consistent with the multivariable analyses results presented in Table 5 and shown in Figure 3A,B.

## 4. Discussion

SGK1 gene expression in PBMCs of MM patients after AHSCT was evaluated for prognostic value in this study. Our findings provide compelling evidence that SGK1 expression dynamics closely parallel disease status and may serve as a novel biomarker for relapse prediction in the post‐transplant setting, with predictive accuracy comparable to, and potentially complementary with, established MRD assessments and sFLC levels.

The AUC from ROC analysis shows that SGK1 gene expression has a great predictive value, which is one of the study’s most important discoveries. SGK1 achieved an AUC of 0.86 for relapse prediction, closely approximating the performance of MFC‐based MRD (AUC 0.88), which is widely regarded as a gold standard in post‐treatment surveillance. This observation underscores the potential of SGK1 as a robust, minimally invasive biomarker that could supplement or even substitute bone marrow‐based MRD in selected clinical contexts. The use of peripheral blood for SGK1 quantification offers a distinct practical advantage, as repeated bone marrow aspirations are invasive, painful, and may be limited by patchy marrow involvement or sampling error.

The observed trend of declining SGK1 expression following AHSCT across all patient groups, particularly in those achieving complete remission, aligns with the hypothesis that SGK1 levels reflect disease burden. This finding is biologically plausible given SGK1’s known involvement in survival pathways, stress responses, and antiapoptotic signaling in malignant cells [17, 18]. In preclinical models, SGK1 has been implicated in conferring resistance to chemotherapy and promoting tumor cell viability via downstream effectors, such as FOXO3a, NF‐κB, and mTORC2 pathways [19–21]. Our clinical data provide a translational extension of these observations, suggesting that SGK1 downregulation post‐transplant may represent a favorable prognostic marker, while sustained or re‐elevated expression may signal impending relapse.

Importantly, the dynamic changes in SGK1 expression were largely concordant with changes in MRD and sFLC levels over time, reinforcing the validity of SGK1 as a marker of disease status. However, in a subset of patients with discordant MRD and SGK1 results, further analysis is warranted to delineate the clinical implications [22]. Notably, SGK1 levels appeared to remain elevated or rebound in certain patients who were MRD‐negative by MFC but subsequently experienced clinical relapse. This raises the possibility that SGK1 may detect early systemic or extramedullary disease not captured by marrow‐based MRD assessments, potentially filling a critical gap in current surveillance strategies.

Another noteworthy observation from this study is the differential expression of SGK1 among cytogenetic risk groups. Individuals with HR features—including t(4;14), del(17p), and 1q21 amplification—demonstrated higher baseline SGK1 expression and a more attenuated decline post‐transplant, in contrast to MR and LR counterparts. These findings suggest that SGK1 expression may be modulated by underlying genetic aberrations that drive disease aggressiveness. While the mechanistic basis of this association remains to be fully elucidated, it is conceivable that HR cytogenetic alterations confer transcriptional or epigenetic changes that upregulate SGK1, thereby promoting chemoresistance and disease persistence [23, 24]. Future studies involving integrative genomic and transcriptomic analyses could provide valuable insights into the regulatory networks linking SGK1 expression and cytogenetic risk stratification in MM.

From a treatment perspective, the prognostic significance of SGK1 expression opens possibilities for risk‐adapted interventions in the post‐transplant setting. Patients with persistently high SGK1 expression may benefit from augmented maintenance strategies that include combination treatments with proteasome inhibitors and immunomodulatory drugs [25]. Moreover, SGK1 may be a therapeutic target itself. Recent preclinical data show that selective inhibitors of SGK1 reduce tumor cell viability and sensitize hematologic malignancies to chemotherapy agents, highlighting their potential as future therapeutic options in MM.

Although these encouraging findings emerged, it is important to recognize limitations. First, as this is a single‐center retrospective study, inherent bias in patient selection and data interpretation should be acknowledged. In addition to the standardized treatment regimen, variability in supportive care, transplant conditioning, and post‐relapse therapies may contribute to variability in the outcomes. Second, a sample size of 85 patients does provide adequate statistical power; future work should aim to provide validation of these results in larger multicenter cohorts. Third, while SGK1 quantification by qRT‐PCR, analytical robustness, and some technical variation will remain when developing standardized procedures, external quality control will be necessary to transition SGK1 analysis into a clinical setting.

Another limitation is the lack of functional studies to establish a cause‐and‐effect relationship between SGK1 expression and relapse. While higher SGK1 expression was correlated with worse outcomes, it cannot be established that SGK1 is a driver of disease progression or a co‐marker. Future studies with gene knock‐out or pharmacological inhibition models to show the causal role of SGK1 in MM biology would help to support SGK1 as a potential therapeutic target. In addition, the exploratory cytokine profiles of PBMCs were not assessed in this study; however, subsequent studies with archived PBMC samples may enable us to explore in the future cytokine signaling, SGK1 downstream targets, and gene expression profiles together.

Although SGK1 expression may vary with cytokine status and stress signals, our data show a consistent prognostic association across risk groups, which supports SGK1’s relative stability as a biomarker. Importantly, SGK1 expression has only slightly lower predictive capacity than MRD, so a model using both MRD and SGK1 expression likely has additive (or even synergistic) value. A multimodal model that includes MRD, sFLC, SGK1 expression, and cytogenetics would likely yield the most accurate prognostication. Machine learning algorithms would be a useful tool for developing such multimodal models and testing their predictive value in future prospective studies.

Lastly, immunomodulation with SGK1 may be an important consideration as we rely more and more on immunotherapies, including monoclonal antibodies, CAR‐T cells, and bispecific T‐cell engagers for treating MM. There is some preliminary evidence that SGK1 may be involved in T‐cell function and immune evasion mechanisms. Therefore, if patients demonstrate persistent SGK1 overexpression post‐transplantation, it may reflect tumor residual disease but also result in impaired immune‐mediated disease control functions that may increase relapse risk. Future research on archived PBMCs could investigate cytokine signaling pathways, SGK1 downstream targets, and the combination with transcriptomic features. Evaluating the immunologic correlates to SGK1 expression could provide more insight into this biomarker’s prognostic value, given the multitude of therapies becoming available to treat patients with MM.

## 5. Conclusion

In summary, our study provides the first clinical evidence supporting SGK1 gene expression as a minimally invasive, dynamic biomarker for relapse prediction in MM patients undergoing AHSCT. SGK1 expression correlates with disease burden, stratifies relapse risk, and offers predictive value comparable to established MRD assessments. Particularly in patients with HR cytogenetics or ambiguous MRD results, SGK1 monitoring may enhance post‐transplant surveillance and inform personalized therapeutic strategies. Further studies in larger, multicenter cohorts are needed to further validate our results, examine SGK1’s mechanistic role in MM pathogenesis, and investigate its feasibility as a therapeutic target in biomarker‐driven clinical trials.

## Author Contributions

Xiangdong Shen is responsible for the guarantor of integrity of the entire study, study concepts and design, literature research, data acquisition and analysis, manuscript preparation, and editing and review. Haiyan Liu is responsible for the guarantor of integrity of the entire study, definition of intellectual content, clinical studies, experimental studies, data analysis, and statistical analysis. Xiaoyu Huang is responsible for the data analysis, manuscript preparation, and editing. Qiaocheng Qiu and Yuhua Ru are responsible for the definition of intellectual content, literature research, clinical studies, and manuscript review. Hui Hui is responsible for the study concepts and design. Juncheng Chen is responsible for the guarantor of integrity of the entire study, study concepts, and definition of intellectual content.

## Funding

The authors have not received any funding support.

## Disclosure

All the authors have read and approved the final manuscript.

## Ethics Statement

This was a study undertaken in compliance with the Declaration of Helsinki. The study was approved by the Affiliated Hospital of Nantong University (Approval Number: 2024‐L020). Written informed consent was obtained from all participants or their legal representatives prior to enrollment.

## Consent

The authors have nothing to report.

## Conflicts of Interest

The authors declare no conflicts of interest.

## Data Availability

The datasets used or analyzed during the current study are available from the responding author upon reasonable request.
